# Supplementary material for: Association of adverse prenatal exposure burden with child psychopathology in the Adolescent Brain Cognitive Development (ABCD) Study
Source: PLoS One. 2021 Apr 28;16(4):e0250235. doi: 10.1371/journal.pone.0250235 (PMC8081164; doi:10.1371/journal.pone.0250235)
Supplement: S1 File — (DOCX) [file pone.0250235.s001.docx]

J.L. Roffman et al.

Association of adverse prenatal exposure burden with child psychopathology in the ABCD study

**Contents**

S1 to S14 Tables pp. 2-15

S1 to S2 Figs pp. 16-17

Methods pp. 18-20

**S1 Table:** Imputation metrics in the Non-sibling group (n=7,898)

| CONTINUOUS FACTORS | % missing, before imputation | Before imputation | | After imputation | |
| --- | --- | --- | --- | --- | --- |
|  |  | Mean | SD | Mean | SD |
| Age (months) | 0.0 | 118.5 | 7.3 | 118.5 | 7.3 |
| CBCL t-score | 0.0 | 46.4 | 11.3 | 46.4 | 11.3 |
| Caregiver education | 0.1 | 16.5 | 2.8 | 16.5 | 2.8 |
| Caregiver income | 9.0 | 7.1 | 2.5 | 7.0 | 2.5 |
| Neighborhood safety | 0.0 | 3.6 | 1.1 | 3.6 | 1.1 |
| Family conflict | 0.0 | 2.5 | 1.9 | 2.5 | 1.9 |
| Screen time - weekdays | 0.1 | 2.4 | 2.7 | 2.4 | 2.7 |
| Screen time - weekend | 0.1 | 3.9 | 2.7 | 3.9 | 2.7 |
| Maternal age - birth | 1.6 | 29.3 | 6.3 | 29.3 | 6.3 |
| DICHOTOMOUS FACTORS | % missing, before imputation | % of sample exposed, before imputation | | % of sample exposed, after imputation | |
| Unplanned pregnancy | 1.2 | 39.3 | | 40.0 | |
| Early alcohol exposure | 4.6 | 26.1 | | 27.6 | |
| Late alcohol exposure | 1.0 | 2.7 | | 2.8 | |
| Early tobacco exposure | 1.1 | 13.8 | | 14.1 | |
| Late tobacco exposure | 0.8 | 5.0 | | 5.0 | |
| Early marijuana expos. | 1.5 | 6.2 | | 6.5 | |
| Late marijuana expos. | 0.9 | 2.0 | | 2.2 | |
| Complicated pregnancy | 3.1 | 39.7 | | 41.0 | |
| Complicated birth | 2.8 | 23.9 | | 24.7 | |
| Pre-term birth | 0.7 | 8.0 | | 8.1 | |
| Caesarian section | 0.4 | 31.7 | | 31.9 | |
| Sex (female) | 0.0 | 46.9 | | 46.9 | |
| Race (non-Caucasian) | 0.1 | 37.7 | | 37.7 | |
| Ethnicity (Latinx) | 1.2 | 22.9 | | 23.3 | |
| First birth | 0.0 | 58.1 | | 58.1 | |
| Trauma exposure | 3.6 | 35.2 | | 36.5 | |
| Partner present | 1.1 | 79.0 | | 79.7 | |

**S2 Table:** Imputation metrics in the Sibling group (n=1,392)

| CONTINUOUS FACTORS | % missing, before imputation | Before imputation | | After imputation | |
| --- | --- | --- | --- | --- | --- |
|  |  | Mean | SD | Mean | SD |
| Age (months) | 0.0 | 118.4 | 8.8 | 118.4 | 8.8 |
| CBCL t-score | 0.0 | 45.1 | 11.3 | 45.1 | 11.3 |
| Caregiver education | 0.3 | 16.2 | 2.9 | 16.2 | 2.9 |
| Caregiver income | 8.8 | 6.8 | 2.6 | 6.8 | 2.6 |
| Neighborhood safety | 0.1 | 3.8 | 1.0 | 3.6 | 1.0 |
| Family conflict | 0.0 | 2.7 | 2.1 | 2.7 | 2.1 |
| Screen time - weekdays | 0.1 | 2.5 | 2.5 | 2.3 | 2.6 |
| Screen time - weekend | 0.1 | 3.8 | 2.6 | 3.7 | 2.6 |
| Maternal age - birth | 1.7 | 27.9 | 5.7 | 27.8 | 5.7 |
| DICHOTOMOUS FACTORS | % missing, before imputation | % of sample exposed, before imputation | | % of sample exposed, after imputation | |
| Unplanned | 0.6 | 41.4 | | 41.5 | |
| Early alcohol | 3.6 | 18.3 | | 19.3 | |
| Late alcohol | 0.8 | 1.4 | | 1.7 | |
| Early tobacco | 0.4 | 11.2 | | 11.3 | |
| Late tobacco | 0.4 | 4.7 | | 4.9 | |
| Early marijuana | 0.5 | 4.4 | | 4.6 | |
| Late marijuana | 0.4 | 2.2 | | 2.4 | |
| Complicated pregnancy | 2.1 | 37.4 | | 37.2 | |
| Complicated birth | 2.2 | 23.6 | | 23.9 | |
| Pre-term birth | 0.6 | 6.9 | | 7.0 | |
| Caesarian section | 0.1 | 26.8 | | 26.8 | |
| Sex (female) | 0.0 | 49.4 | | 49.4 | |
| Race (non-Caucasian) | 0.3 | 36.2 | | 36.3 | |
| Ethnicity (Latinx) | 0.0 | 19.7 | | 20.4 | |
| First birth | 0.0 | 38.6 | | 38.6 | |
| Trauma exposure | 4.5 | 34.0 | | 34.3 | |
| Partner present | 1.4 | 82.6 | | 81.8 | |

**S3 Table:** Correlations among in the Non-sibling group (n=7,898), controlled for site

| ***p<.05**  ****p<.001**  CG=caregiver | Unplanned pregnancy | Early alcohol | Late alcohol | Early tobacco | Late tobacco | Early marijuana | Late marijuana | Complicat. pregnancy | Complicat. birth | Pre-term birth | Caesarian section |
| --- | --- | --- | --- | --- | --- | --- | --- | --- | --- | --- | --- |
| Unplanned pregnancy |  |  |  |  |  |  |  |  |  |  |  |
| Early alcohol | **  .078 |  |  |  |  |  |  |  |  |  |  |
| Late  alcohol | -.006 | **  .238 |  |  |  |  |  |  |  |  |  |
| Early tobacco | **  .199 | **  .185 | **  .050 |  |  |  |  |  |  |  |  |
| Late tobacco | **  .133 | **  .066 | **  .102 | **  .510 |  |  |  |  |  |  |  |
| Early marijuana | **  .184 | **  .189 | **  .085 | **  .330 | **  .187 |  |  |  |  |  |  |
| Late marijuana | **  .112 | **  .108 | **  .161 | **  .201 | **  .188 | **  .525 |  |  |  |  |  |
| Complicat. pregnancy | **  .058 | -.002 | -.018 | **  .065 | **  .054 | **  .0942 | **  .043 |  |  |  |  |
| Complicat. birth | *  .022 | *  .024 | .003 | **  .052 | *  .032 | *  .023 | .016 | **  .111 |  |  |  |
| Pre-term birth | .017 | -.008 | -.007 | .017 | *  .033 | .006 | .010 | **  .158 | **  .232 |  |  |
| Caesarian section | -.036 | *  .028 | .016 | .003 | .009 | -.017 | -.020 | **  .100 | **  .047 | **  .080 |  |
| Age | .011 | -.010 | -0.20 | .038 | .022 | -.018 | -.011 | *  -.015 | .004 | -.001 | .003 |
| Sex (female) | *  .029 | .022 | .013 | -.013 | .013 | .015 | *  .029 | *  .023 | **  -.055 | -.020 | *  -.049 |
| Race (non-Cauc.) | **  .218 | **  -.130 | **  -.074 | **  .041 | .010 | **  .055 | *  .011 | **  .078 | .004 | -.002 | *  -.045 |
| Ethnicity (Latinx) | **  -.098 | **  .057 | *  .027 | .004 | *  .024 | -.010 | .014 | **  -.033 | *  .002 | -.014 | -.006 |
| CG educ. | **  -.261 | **  .152 | **  .057 | **  -.203 | **  -.154 | **  -.113 | **  -.071 | **  -.092 | .003 | .001 | .037 |
| CG income | **  -.310 | **  .165 | **  .060 | **  -.185 | **  -.134 | **  -.148 | **  -.099 | **  -.112 | .005 | -.010 | .030 |
| CG partner (no) | **  .215 | -.039 | *  -.032 | **  .099 | **  .067 | **  .091 | **  .048 | **  .053 | .016 | .012 | *  -.032 |
| First birth (yes) | **  .095 | **  .119 | *  .032 | **  .101 | **  .051 | **  .102 | **  .051 | **  .056 | **  .087 | *  .037 | .006 |
| Neighborh’d safety | **  -.156 | .034 | -.004 | **  -.094 | **  -.069 | **  -.103 | **  -.074 | **  -.085 | *  -.044 | -.007 | .008 |
| Family conflict | **  .068 | **  .067 | **  .045 | **  .081 | **  .049 | **  .090 | **  .057 | **  .027 | *  .016 | -.002 | .008 |
| Screentime - weekday | **  .138 | *  -.032 | -.018 | **  .099 | **  .063 | **  .067 | **  .080 | *  .044 | -.015 | .028 | .000 |
| Screentime - weekend | **  .132 | .004 | -.009 | **  .112 | **  .094 | **  .080 | **  .057 | **  .064 | .009 | .013 | .023 |
| Trauma exposure | **  .120 | **  .048 | *  .032 | **  .085 | **  .068 | **  .091 | **  .073 | **  .079 | **  .058 | *  .029 | -.017 |
| Mat. age at birth | **  -.277 | **  .063 | **  .062 | **  -.177 | **  -.099 | **  -.159 | **  -.084 | **  -.038 | -.008 | .016 | **  .135 |

**S4 Table:** Effect of each individual exposure on CBCL total score in the imputed Non-sibling group (n=7,898), adjusted only for site and late alcohol, tobacco, or marijuana exposure (minimally adjusted)

| EXPOSURE | CBCL total,  unexposed group | CBCL total,  exposed group | p |
| --- | --- | --- | --- |
|  | Mean (95% CI) | Mean (95% CI) |  |
| Unplanned pregnancy | 51.1 (49.8 to 52.4) | 52.6 (51.3 to 53.9) | <.001 |
| Early alcohol exposure | 51.2 (49.9 to 52.5) | 52.5 (51.2 to 53.8) | <.001 |
| Early tobacco exposure | 50.4 (49.1 to 51.8) | 53.2 (51.9 to 54.6) | <.001 |
| Early marijuana expos. | 50.8 (49.3 to 52.2) | 52.9 (51.5 to 54.3) | .001 |
| Complicated pregnancy | 50.2 (48.9 to 51.5) | 53.4 (52.2 to 54.7) | <.001 |
| Complicated birth | 50.7 (49.4 to 51.9) | 53.0 (51.7 to 54.3) | <.001 |
| Preterm birth | 51.8 (50.6 to 53.0) | 51.8 (50.4 to 53.3) | .961 |
| Caesarian section | 51.8 (50.5 to 53.1) | 51.9 (50.6 to 53.2) | .761 |

**S5 Table:** Effect of each individual exposures on CBCL total score in the imputed Non-sibling group (n=7,898), fully adjusted

| EXPOSURE | CBCL total,  unexposed group | CBCL total,  exposed group | p |
| --- | --- | --- | --- |
|  | Mean (95% CI) | Mean (95% CI) |  |
| Unplanned pregnancy | 50.4 (49.1 to 51.7) | 51.5 (50.1 to 52.8) | <.001 |
| Early alcohol exposure | 50.2 (48.8 to 51.5) | 51.7 (50.4 to 53.0) | <.001 |
| Early tobacco exposure | 49.8 (48.4 to 51.1) | 52.1 (50.7 to 53.4) | <.001 |
| Early marijuana expos. | 50.2 (48.7 to 51.6) | 51.7 (50.3 to 53.1) | .017 |
| Complicated pregnancy | 49.4 (48.1 to 50.8) | 52.4 (51.1 to 53.7) | <.001 |
| Complicated birth | 49.9 (48.5 to 51.2) | 52.0 (50.7 to 53.3) | <.001 |
| Preterm birth | 50.9 (49.7 to 52.2) | 50.9 (49.5 to 52.4) | .943 |
| Caesarian section | 50.9 (49.6 to 52.2) | 50.9 (49.6 to 52.3) | .893 |

**S6 Table:** Effect of individual exposures on CBCL total score in the non-imputed Non-sibling group (n=6,271), fully adjusted

| EXPOSURE | CBCL total,  unexposed group | CBCL total,  exposed group | p |
| --- | --- | --- | --- |
|  | Mean (95% CI) | Mean (95% CI) |  |
| Unplanned pregnancy | 49.5 (48.0 to 51.0) | 50.3 (48.8 to 51.8) | .012 |
| Early alcohol exposure | 49.1 (47.6 to 50.7) | 50.6 (49.1 to 52.1) | <.001 |
| Early tobacco exposure | 48.8 (47.2 to 50.4) | 51.0 (49.4 to 52.5) | <.001 |
| Early marijuana expos. | 49.2 (47.5 to 50.8) | 50.6 (49.0 to 52.3) | .036 |
| Complicated pregnancy | 48.5 (47.0 to 50.0) | 51.3 (49.8 to 52.8) | <.001 |
| Complicated birth | 48.9 (47.4 to 50.4) | 50.9 (49.4 to 52.4) | <.001 |
| Preterm birth | 49.8 (48.4 to 51.3) | 50.0 (48.3 to 51.6) | .800 |
| Caesarian section | 49.9 (48.4 to 51.4) | 49.9 (48.4 to 51.4) | .892 |

**S7 Table:** Odds of CBCL total score ≥60 for individual exposures in the imputed Non-sibling group (n=7,898)

| EXPOSURE | Odds of CBCL total ≥60 (minimally adjusted) | | Odds of CBCL total ≥60  (fully adjusted) | |
| --- | --- | --- | --- | --- |
|  | Odds ratio (95% CI) | p | Odds ratio (95% CI) | p |
| Unplanned pregnancy | 1.59 (1.38 to 1.83) | <.001 | 1.35 (1.16 to 1.58) | <.001 |
| Early alcohol exposure | 0.99 (0.84 to 1.17) | .938 | 1.12 (0.94 to 1.33) | .215 |
| Early tobacco exposure | 1.77 (1.43 to 2.19) | <.001 | 1.56 (1.25 to 1.94) | <.001 |
| Early marijuana expos. | 1.32 (0.98 to 1.76) | .064 | 1.16 (0.86 to 1.56) | .321 |
| Complicated pregnancy | 1.71 (1.48 to 1.97) | <.001 | 1.63 (1.42 to 1.89) | <.001 |
| Complicated birth | 1.56 (1.34 to 1.81) | <.001 | 1.54 (1.32 to 1.80) | <.001 |
| Preterm birth | 0.99 (0.78 to 1.26) | .949 | 1.01 (0.79 to 1.28) | .948 |
| Caesarian section | 1.01 (0.87 to 1.16) | .934 | 0.99 (0.86 to 1.15) | .944 |

**S8 Table:** Effect of adverse prenatal exposure load on CBCL total score in the initial (non-Sibling) group (n=7,898)

| **EXPOSURE LOAD, N** | **Effect on CBCL Total**  **(minimally adjusted)** | | **Effect on CBCL Total**  **(fully adjusted)** | |
| --- | --- | --- | --- | --- |
|  | **Estimate (95% CI)** | **p** | **Estimate (95% CI)** | **p** |
| 0, N=1,640 | Reference | -- | Reference | -- |
| 1, N=2,712 | 1.56 (0.88 to 2.24) | <.001 | 1.25 (0.58 to 1.92) | <.001 |
| 2, N=1,985 | 4.19 (3.46 to 4.92) | <.001 | 3.70 (2.97 to 4.43) | <.001 |
| 3, N=994 | 6.45 (5.55 to 7.35) | <.001 | 5.65 (4.75 to 6.56) | <.001 |
| ≥4, N=567 | 9.22 (8.06 to 10.37) | <.001 | 8.16 (6.99 to 9.32) | <.001 |
| Linear effect of load | 2.18 (1.97 to 2.40) | <.001 | 1.94 (1.72 to 2.16) | <.001 |

**S9 Table:** Effects of exposure loading on odds of elevated CBCL syndrome (t score ≥65) and broad-band (t score ≥60) scores in the imputed Non-sibling group (n=7,898, fully adjusted)

| CBCL SCALE | N above normal | Linear effect of load | | 1 vs 0 exposures | | | 2 vs 0 exposures | | | 3 vs 0 exposures | | | ≥4 vs 0 exposures | | |
| --- | --- | --- | --- | --- | --- | --- | --- | --- | --- | --- | --- | --- | --- | --- | --- |
|  |  | ß | *p | OR | 95% CI | p | OR | 95% CI | p | OR | 95% CI | p | OR | 95% CI | p |
| Syndrome Scales | | | | | | | | | | | | | | | |
| Anxious/  Depressed | 540 | 0.19 | <.001 | 0.89 | 0.69-1.16 | .403 | 1.28 | 0.98-1.67 | .067 | 1.68 | 1.25-2.28 | .001 | 1.98 | 1.38-2.82 | <.001 |
| Withdrawn/  Depressed | 588 | 0.18 | <.001 | 1.00 | 0.75-1.32 | .980 | 1.31 | 0.98-1.74 | .071 | 1.71 | 1.23-2.36 | .001 | 1.83 | 1.25-2.67 | .002 |
| Somatic  Complaints | 705 | 0.24 | <.001 | 1.36 | 1.04-1.78 | .023 | 1.73 | 1.31-2.28 | <.001 | 2.37 | 1.75-3.22 | <.001 | 2.83 | 1.98-4.04 | <.001 |
| Social  Problems | 244 | 0.32 | <.001 | 1.07 | 0.69-1.67 | .750 | 2.00 | 1.31-3.05 | .001 | 3.21 | 2.06-5.02 | <.001 | 3.26 | 1.97-5.41 | <.001 |
| Thought  Problems | 666 | 0.27 | <.001 | 1.08 | 0.81-1.44 | .587 | 1.72 | 1.30-2.88 | <.001 | 2.09 | 1.52-2.87 | <.001 | 2.90 | 2.04-4.13 | <.001 |
| Attention  Problems | 631 | 0.31 | <.001 | 1.15 | 0.85-1.54 | .367 | 1.86 | 1.39-2.49 | <.001 | 2.71 | 1.98-3.73 | <.001 | 3.30 | 2.29-4.75 | <.001 |
| Rule-breaking  Behavior | 337 | 0.34 | <.001 | 0.83 | 0.52-1.31 | .425 | 1.93 | 1.27-2.95 | .002 | 2.62 | 1.67-4.11 | <.001 | 3.30 | 2.01-5.44 | <.001 |
| Aggressive  Behavior | 355 | 0.34 | <.001 | 0.97 | 0.68-1.40 | .878 | 1.70 | 1.19-2.42 | .003 | 3.02 | 2.08-4.38 | <.001 | 3.48 | 2.28-5.33 | <.001 |
| Broad Band Scales | | | | | | | | | | | | | | | |
| Internalizing  Problems | 1287 | 0.25 | <.001 | 1.22 | 1.01-1.47 | .039 | 1.61 | 1.33-1.96 | <.001 | 2.16 | 1.72-2.70 | <.001 | 2.83 | 2.17-3.69 | <.001 |
| Externalizing  Problems | 703 | 0.28 | <.001 | 0.96 | 0.74-1.24 | .764 | 1.46 | 1.13-1.89 | .004 | 2.23 | 1.68-2.95 | <.001 | 2.76 | 2.00-3.82 | <.001 |

*P-values are unadjusted for multiple comparisons; all linear load analyses remain significant at p<.05 after controlling for ten comparisons using the family-wise error rate.

**S10 Table:** Effect of adverse prenatal exposure load on CBCL total score in the validation (Sibling) group (n=1,392)

| **EXPOSURE LOAD, N** | **Effect on CBCL Total**  **(minimally adjusted)** | | **Effect on CBCL Total**  **(fully adjusted)** | |
| --- | --- | --- | --- | --- |
|  | **Estimate (95% CI)** | **p** | **Estimate (95% CI)** | **p** |
| 0, N=353 | Reference | -- | Reference | -- |
| 1, N=478 | 1.83 (0.41 to 3.26) | .011 | 1.77 (0.36 to 3.20) | .014 |
| 2, N=343 | 3.74 (2.14 to 5.34) | <.001 | 3.61 (2.00 to 5.21) | <.001 |
| 3, N=143 | 6.45 (4.31 to 8.58) | <.001 | 6.29 (4.14 to 8.44) | <.001 |
| ≥4, N=75 | 7.57 (4.62 to 10.53) | <.001 | 7.42 (4.43 to 10.41) | <.001 |
| Linear effect of load | 1.90 (1.35 to 2.45) | <.001 | 1.86 (1.31 to 2.42) | <.001 |

**S11 Table:** Effect of adverse prenatal exposure load on CBCL total score in the validation (Sibling) group (n=1,392) without including Family ID as a covariate

| **EXPOSURE LOAD, N** | **Effect on CBCL Total**  **(minimally adjusted)** | | **Effect on CBCL Total**  **(fully adjusted)** | |
| --- | --- | --- | --- | --- |
|  | **Estimate (95% CI)** | **p** | **Estimate (95% CI)** | **p** |
| 0, N=353 | Reference | -- | Reference | -- |
| 1, N=478 | 2.19 (0.66 to 3.72) | .005 | 2.17 (0.64 to 3.69) | .005 |
| 2, N=343 | 4.98 (3.32 to 6.64) | <.001 | 4.77 (3.12 to 6.44) | <.001 |
| 3, N=143 | 8.09 (5.86 to 10.33) | <.001 | 8.16 (5.92 to 10.41) | <.001 |
| ≥4, N=75 | 9.38 (6.28 to 12.49) | <.001 | 9.42 (6.30 to 12.54) | <.001 |
| Linear effect of load | 2.44 (1.88 to 2.99) | <.001 | 2.44 (1.88 to 3.00) | <.001 |

**S12 Table:** Effect of adverse prenatal exposure load on odds of CBCL total score ≥60 in the validation (Sibling) group (n=1,392) without including Family ID as a covariate

| **EXPOSURE LOAD, N** | **Odds of CBCL total ≥60**  **(minimally adjusted)** | | **Odds of CBCL total ≥60**  **(fully adjusted)** | |
| --- | --- | --- | --- | --- |
|  | **Odds ratio (95% CI)** | **p** | **Odds ratio (95% CI)** | **p** |
| 0, N=353 | Reference | -- | Reference | -- |
| 1, N=478 | 2.28 (1.18 to 4.39) | .014 | 2.12 (1.09 to 4.13) | .027 |
| 2, N=343 | 3.96 (2.06 to 7.58) | <.001 | 3.39 (1.74 to 6.60) | <.001 |
| 3, N=143 | 6.51 (3.16 to 13.41) | <.001 | 5.48 (2.60 to 11.56) | <.001 |
| ≥4, N=75 | 8.06 (3.42 to 18.98) | <.001 | 6.82 (2.79 to 16.72) | <.001 |
| Linear effect of load | 1.62 (1.38 to 1.90) | <.001 | 1.57 (1.32 to 1.85) | <.001 |

**S13 Table:** Effect of adverse prenatal exposure load on odds of CBCL total score ≥60 in the initial (Nonsibling) group (n=7,898), additionally including postnatal exposures (screen time, traumatic exposure, parental conflict)

| **EXPOSURE LOAD, N** | **Odds of CBCL total ≥60** | |
| --- | --- | --- |
|  | **Odds ratio (95% CI)** | **p** |
| 0, N=1,640 | Reference | -- |
| 1, N=2,712 | 1.10 (0.86 to 1.40) | .459 |
| 2, N=1,985 | 1.59 (1.24 to 2.02) | <.001 |
| 3, N=994 | 2.27 (1.74 to 2.97) | <.001 |
| ≥4, N=567 | 2.53 (1.85 to 3.46) | <.001 |

**S14 Table:** Effect of adverse prenatal exposure load on odds of CBCL total score ≥60 in the validation (Sibling) group (n=1,392), additionally including postnatal exposures (screen time, traumatic exposure, parental conflict)

| **EXPOSURE LOAD, N** | **Odds of CBCL total ≥60** | |
| --- | --- | --- |
|  | **Odds ratio (95% CI)** | **p** |
| 0, N=353 | Reference | -- |
| 1, N=478 | 1.88 (0.90 to 3.91) | .093 |
| 2, N=343 | 2.73 (1.30 to 5.75) | .008 |
| 3, N=143 | 3.55 (1.49 to 8.45) | .004 |
| ≥4, N=75 | 4.16 (1.44 to 12.02) | .008 |

**S1 Fig:** Participant selection based on inclusion and exclusion criteria for the current analysis

**S2 Fig:** Effect of sibling discordance for adverse prenatal exposure load on CBCL total score in the Sibling group, after controlling for age, sex, maternal age at birth, and family-average exposure load

**Methods**

Demographics survey: Primary caregiver education was coded as follows and treated as a scalar variable in fully adjusted models.

0 = Never attended/Kindergarten only
1 = 1st grade

2 = 2nd grade

3 = 3rd grade

4 = 4th grade

5 = 5th grade

6 = 6th grade

7 = 7th grade

8 = 8th grade

9 = 9th grade

10 = 10th grade

11 = 11th grade

12 = 12th grade

13 = High school graduate

14 = GED or equivalent

15 = Some college

16 = Associate degree: Occupational

17 = Associate degree: Academic Program

18 = Bachelor's degree

19 = Master's degree

20 = Professional School degree

21 = Doctoral degree

Family income was coded as follows and treated as a scalar variable in fully adjusted models.

1=Less than 5,000

2=5,000-11,999

3=12,000-15,999

4=16,000-24,999

5=25,000-34,999

6=35,000-49,999

7=50,000-74,999

8=75,000-99,999

9=100,000-199,999

10=200,000+

Developmental history questionnaire: Presence of pregnancy complications was coded as “yes” if at least one of the following was endorsed.

- Severe nausea and vomiting extending past the 6^th^ month or accompanied by weight loss
- Heavy bleeding requiring bed rest or special treatment
- Pre-eclampsia, eclampsia, or toxemia
- Severe gall bladder attack
- Persistent proteinuria
- Rubella during first 3 months of pregnancy
- Severe anemia
- UTI
- Pregnancy-related diabetes
- Pregnancy-related high blood pressure
- Previa, abruptio, other problems with placenta
- Accident or injury requiring medical care
- Any other conditions requiring medical care

Presence of birth complications was coded as “yes” if at least one of the following was endorsed.

- Blue at birth
- Slow heart beat
- Did not breathe at first
- Convulsions
- Jaundice needing treatment
- Required oxygen
- Required blood transfusion
- Rh incompatibility

K-SADS trauma exposure: Presence of the postnatal trauma exposure for the child was coded as “yes” if at least of the following was endorsed.

- A car accident in which your child or another person in the car was hurt bad enough to require medical attention
- Another significant for which your child needed specialized and intensive medical treatment
- Witnessed or caught in a fire that caused significant property damage or personal injury
- Witnessed or caught in a natural disaster that caused significant property damage or personal injury
- Witnessed or present during an act of terrorism
- Witnessed death or mass destruction in a war zone
- Witnessed someone shot or stabbed in the community
- Shot, stabbed, or beaten brutally by a non-family member
- Shot, stabbed, or beaten brutally by a grown up in the home
- Beaten to the point of having bruises by a grown up in the home
- A non-family member threatened to kill your child
- A family member threatened to kill your child
- Witness the grownups in the home push, shove or hit one another
- A grownup in the home touched your child in his or her privates, had your child touch their privates, or did other sexual things to your child
- An adult outside your family touched your child in his or her privates, had your child touch their privates or did other sexual things to your child
- A peer forced your child to do something sexually
- Learned about the sudden unexpected death of a loved one
