# Supplementary material for: Association of adverse prenatal exposure burden with child psychopathology in the Adolescent Brain Cognitive Development (ABCD) Study
Source: PLoS One. 2021 Apr 28;16(4):e0250235. doi: 10.1371/journal.pone.0250235 (PMC8081164; doi:10.1371/journal.pone.0250235)
Supplement: Attachment — Submitted filename: Response_to_review_R2_forsubmission_032321.docx [file pone.0250235.s003.docx]

**Response to Reviewers (second round of review)**

**Reviewer #1**

**The authors have done a great job addressing most of my concerns. I have some additional comments/minor suggestions:**

Thank you for your additional comments which have helped us to refine the manuscript.

**1. Introduction:
Page 5 (line 101) you write “risk for significant psychopathology” – the word significant should be deleted.**

Done – deleted “significant.”

**2. Page 6 (line 133) – you write “incompletely expressed psychopathology” – unclear what this means, please rewrite.**

Replaced “incompletely expressed” with “subdiagnostic.”

**3. Page 6 (line 139) – “individual- and family-level variables” – delete individual?**

Done – deleted “individual.”

**4. Page 6 (line 139-141)- I would recommending rewriting the last section on page 6 to something like: “If the association is causal, siblings who are discordant for exposures should show greater differences in psychopathology. Conversely, if the discordant siblings do not differ in psychopathology, the observed association is explained by unmeasured genetic and environmental factors. The results of studies with sibling design have supported causal relationships between prenatal exposure to tobacco (40), alcohol (41), and obstetrical complications (42).”
Note: Regarding tobacco, results are conflicting, please see: https://doi.org/10.1542/peds.2016-2509**

We appreciate the reviewer’s suggested edits and have incorporated them wholesale (bottom of p.5/top of p.6). In regard to tobacco, we agree that results from previous sibling studies have been inconsistent and now indicate this more clearly in the discussion (citation 51 is the Gustavson paper that the reviewer specifically mentioned):

“Despite the smaller sample size for that analysis, this finding suggests that the relationship between prenatal exposure load and CBCL is unlikely to be confounded by unmeasured family-level differences, a possibility suggested by previous work ( e.g., (19, 51)).” (middle of p.21)

**Methods:
5. Exposures: In line with comments from reviewer 2 I would recommend rethinking the variable selection procedure.**

Thank you for this comment. In the previous round of review, Reviewer 2 questioned how we selected the adverse exposures that were subsequently entered into the exposure load analysis, and specifically suggested that we use lasso regression and elastic net.

Rather, we had used multivariate regression to select exposures for additive analyses, reflecting that [1] each of the included individual exposures had been previously associated with adverse neurodevelopmental outcomes in previous studies, as well as here; [2] this approach enabled development of a clinically intuitive and straightforward risk score analysis that aggregated known risk factors while accounting for overlapping variance; and [3] given the sample size there were no concerns about overfitting and therefore no need to reduce the number of dimensions.

While there are certainly numerous potential approaches to variable selection, feel that the approach we took is appropriate and well justified, and note that Reviewer 2 found our response satisfactory in the last round of review. We therefore elected to keep this approach in the current revision, but now provide a clearer justification for this approach within the manuscript.

As now described more explicitly in the Methods (bottom of p.14/top of p.15):

“Finally, to determine the cumulative odds of CBCL total ≥60 as a function of loading for adverse prenatal exposures, the GLMM was repeated, substituting the integer sum of a child’s prenatal exposures; this loading score was calculated using only exposures that were shown to associate with increased CBCL total scores in the fully adjusted multivariate regression models (above). We used this approach to generate additive exposure loading scores because [1] each of the included individual exposures had been previously associated with adverse neurodevelopmental outcomes in previous studies (as well as in this one); [2] it enabled development of a clinically intuitive and straightforward risk score analysis that aggregated known risk factors while accounting for overlapping variance; and [3] given the sample size there were no concerns about overfitting and therefore no need to reduce the number of dimensions.”

**6. Covariates: I still think that covariates should be restricted to those that are related to both exposures and outcome. I suggest the new sensitivity analyses should be the main analyses (Tables S13, S14). Possible consequences and mediator effects can be investigated in separate supplementary analyses.**

We have implemented this suggestion, so the primary (“fully adjusted”) analysis now includes only covariates that are related to exposures and outcome (and, specifically, postnatal exposure to trauma, parental conflict, and screen time are now excluded).

As a result of this change, an additional prenatal exposure, early marijuana use, became newly significant in the first-level analysis (which identified risk factors that contributed unique variance to CBCL total score). This brought the total number of such exposures to 6. Accordingly, 6-factor load score was carried forward to all subsequent analyses.

The new results are effectively unchanged. There continue to be highly significant effects of adverse prenatal exposure load on CBCL scores, measured either as a dimensional or dichotomous outcome, in the initial (non-sibling) group; these effect replicate in the validation (sibling) group; and siblings who are discordant for exposure load continue to demonstrate differences in CBCL scores, where the sibling with the larger number of exposures exhibited significantly higher CBCL total score.

Per the reviewer’s suggestion we now include models that are additional adjusted for postnatal exposures as sensitivity analyses (S13 and S14).

**Discussion:
7. It is stated (in the response to reviewer #2) that the ABCD study was not designed to be a representative study of the US population. I am curious – what was it designed to be? To what population can the results then be generalized?**

We thank the reviewer for mentioning this point and now address it more completely. The ABCD Study was designed to be geographically diverse (21) and “generally” representative of the demographic and socioeconomic diversity of the US. However, that enrollment was based largely at academic/urban centers could introduce selection bias, and as such it is not considered “representative” of the US population in ways that other datasets (e.g., American Community Survey) might be.

Accordingly, we added the following text to the limitations section of the Discussion:

“[N]otably, though, as the ABCD study recruited from academic centers, urban/suburban populations are better represented, and results may not generalize as well to rural communities.” (p. 25)

**Reviewer #2**

**The authors have revised their manuscript well. They have followed most recommendations and given sound reasons when not. I do not have any further comments.**

Thank you.
